# On the Design of Soret Zone Plates Based on Binary Sequences Using Directional Transducers

**DOI:** 10.3390/s21186086

**Published:** 2021-09-10

**Authors:** Pilar Candelas, Sergio Pérez-López, José Miguel Fuster

**Affiliations:** Centro de Tecnologías Físicas, Universitat Politècnica de València, 46022 València, Spain; pcandelas@fis.upv.es (P.C.); serpelo1@teleco.upv.es (S.P.-L.)

**Keywords:** zone plate, binary sequence, cantor set, fractal, acoustic focusing

## Abstract

In this work, we analyze the effect of the distribution of transparent Fresnel regions over the focusing profile of Soret Zone Plates (SZP) based on binary sequences. It is shown that this effect becomes very significant in those fields where directional transducers are employed, such as microwaves or acoustics. A thorough analysis of both the SZP transmission efficiency and the focusing enhancement factor is presented. Moreover, experimental measurements are also carried out for a particular type of binary sequence, the Cantor ternary set, validating the theoretical model and demonstrating that the distribution of transparent Fresnel regions becomes a critical parameter in applications requiring directional emitters.

## 1. Introduction

Acoustic waves are employed in a wide range of fields because they are able to penetrate deep into objects or biological tissues where optical techniques are not feasible due to scattering and absorption. In particular, ultrasound focusing is widely employed in non-destructive testing in industrial scenarios [1] and biomedical imaging in medical applications [2]. In the past few years, a wide range of novel methods to focus ultrasound waves have been devised. For instance, holographic lenses allow us to focus the acoustic intensity into complex arbitrary 3D shapes by using a simple 3D printed phase-plate [3,4,5,6]. On the other hand, acoustic metamaterials and metasurfaces can focus the incident energy by shaping the incident wavefront into targeted complex amplitude and phase pressure contributions, with each one of their unit cells. These unit cells are usually designed by using Helmholtz resonators [7], subwavelength slits [8], or coiling-up space channels [9,10,11]. Despite the high versatility and focusing capabilities of holographic lenses and acoustic metasurfaces, these kind of structures usually require a complex design process with optimization algorithms and 3D wave propagation numerical simulations to achieve the desired pressure pattern.

In contrast, Fresnel Zone Plates (FZPs) are planar monofocal lenses that can achieve a good focusing performance while maintaining an easy design and manufacturing process. This type of lenses have been widely employed since decades ago in various fields, including microwaves [12], optics [13,14], and acoustics [15,16,17]. FZPs are built by concatenating a series of concentric rings of decreasing width. Each ring is known as a Fresnel region, and its width and inner and outer radii depend on various parameters, such as the focal distance and the operating frequency of the lens. The different Fresnel regions can be implemented by alternating either pressure blocking (opaque) with transparent regions, which is known as a Soret zone plate (SZP), or by alternating phase-reversal with transparent regions, which results in a Phase-Reversal zone plate.

Interestingly, in recent years, novel kinds of lenses based on applying a binary sequence to the different Fresnel regions of a conventional FZP [18] have been introduced. Depending on the nature of this binary mask, different focusing properties are obtained. For instance, using a Fibonacci binary mask results in a bifocal profile with two equal-intensity foci [19,20,21], while using fractal Cantor sequences provides a focusing profile with interesting self-similarity multifocal properties [22,23,24,25]. Thue–Morse binary sequences combine both effects and provide focusing profiles with bifocal fractal properties [26,27]. Thus, the use of binary sequences is very appealing in the design of SZPs, because they provide high versatility in the design of the focusing profile, allowing us to tailor it to the required application. In optics, fractal structures were previously analyzed due to their self-similarity properties in the lateral and axial directions [28,29].

It has already been shown that the distribution of the transparent Fresnel regions on a conventional Soret FZP affects both the transmission and the focusing efficiencies of the lens when directional emitters are involved [30], demonstrating that, when the central region is transparent, higher efficiencies are achieved. In this work, we are going to extend this research to any kind of SZP based on binary sequences [18].

## 2. Theoretical Model

### 2.1. Soret Zone Plates Implemented with Binary Sequences

In plane wave incidence scenarios, which is usually the case in most optical applications, the difference between Soret FZPs with opaque and transparent central regions is only significant for a small number of Fresnel regions as was previously shown in [30]. When the number of Fresnel regions increases, the transmission efficiency of both lenses tends to 50%, and both types of lenses perform identically. Therefore, in optics, where hundreds of Fresnel regions are usually employed, the decision of the central region type becomes irrelevant, as long as there is plane wave incidence. In contrast, in many situations, real underwater ultrasound applications require the use of a directional emitter, such as a piston transducer, and therefore neither the amplitude nor the phase of the incident pressure field is plane. These emitters present a directivity pattern with a main lobe and several secondary lobes [17]. Thus, the contributions of the different Fresnel zones across the surface of the SZP are in this case weighted by the directivity pattern of the transducer, and the inner areas contribute more significantly to the focus than the outer areas. Therefore, the design decision regarding whether the central Fresnel area is transparent or opaque becomes critical, and can significantly modify the focusing performance of the lens.

In a conventional Soret FZP, the transparent and opaque areas are distributed in a sandwich pattern, with each transparent region located between two opaque regions and vice versa. However, there is still one design decision that becomes very significant: the determination of the central Fresnel region. If the central Fresnel region is opaque (O-FZP), the rest of the Fresnel regions are already determined as well. The even-order Fresnel regions are transparent, whereas the odd-order Fresnel regions are opaque, as is the central one (first Fresnel region). On the contrary, if the central Fresnel region is transparent (T-FZP), the even-order and odd-order regions are then reversely opaque and transparent, respectively. As we stated before, the inner regions are favored in power distribution due to the directivity diagram of the piston transducer. Therefore, common sense indicates that the T-FZP lens will be more efficient than the O-FZP lens. This is very straightforward because the number or transparent and opaque regions in an FZP is basically identical and, moreover, these regions are very evenly distributed. For example, the binary sequence of length L=27 for a conventional FZP, would be {101010101010101010101010101} for the O-FZP lens, and {010101010101010101010101010} for the T-FZP case. In these binary sequences, a “1” indicates that the corresponding Fresnel region is opaque, whereas a “0” corresponds to a transparent Fresnel region, and the first value of the binary sequence corresponds to the central Fresnel region.

However, if a different type of binary sequence is used, for example, a Cantor binary sequence, the number of transparent and opaque regions may not be as evenly distributed as in the conventional FZP case. For instance, if the Cantor ternary set of order S=3 is considered, the resulting binary sequence is {010111010111111111010111010} with length L=27 when considering a transparent central Fresnel region (T-CZP). In this case, it is not as clear as in the conventional FZP case whether a transparent central Fresnel region is the right choice for the design of the Cantor Zone Plate (CZP). Although the first region is transparent, there are only a total of 8 transparent regions in the T-CZP, while the total number of opaque regions add up to 19. On the contrary, if the CZP with an opaque central Fresnel region (O-CZP) is considered, the number of transparent and opaque regions is now reversed, and corresponds to 19 and 8, respectively. The binary sequence in this case would be {101000101000000000101000101}. Although the first Fresnel region is now opaque, there are many more transparent than opaque regions, and the design decision to improve the focusing efficiency is not as clear as in the conventional FZP case. Table 1 shows the binary sequences corresponding to the first three orders (S=1,2,3) of the Cantor ternary set, as well as the conventional FZP binary sequences of the same length. All sequences are shown for both the T-SZP and O-SZP cases. As can be observed from Table 1, the binary sequence for the Cantor ternary set of order S=1 matches the FZP binary sequence of the same length because it is a very short binary sequence (L=3). However, when the order of the Cantor binary sequence increases, the binary sequence becomes larger, and the CZP and FZP binary sequences become much more distinct. The same argument can be stated for other type of binary sequences, such as Mbonacci, Thue–Morse or predistorted sequences. Table 2 shows some binary sequence examples corresponding to different O-SZPs. For clarification purposes, FZP refers to a conventional Soret Fresnel zone plate, CZP corresponds to a Soret Cantor zone plate and SZP refers to any kind of Soret zone plate, FZP and CZP included. Furthermore, the prefixes T- and O- can be indistinctly applied before any kind of zone plate: FZP, CZP or SZP.

**Figure 1:**
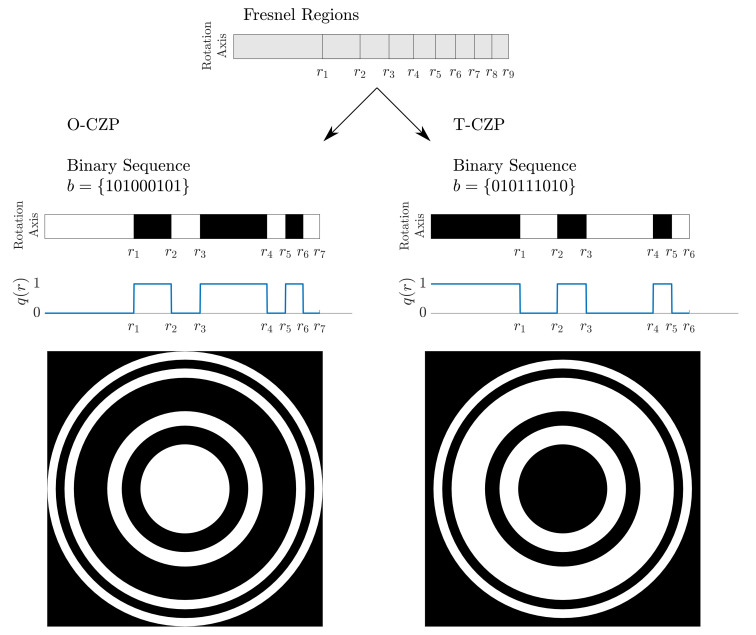
Cantor ZP design example using binary sequences for the opaque central region (O-CZP) and transparent central region (T-CZP) cases. From top to bottom: Fresnel region distribution for the L=9 case, schematic view of the activation procedure of the Fresnel regions using the governing binary sequence for both O-CZP and T-CZP cases, pupil function q(r), and final ZP layouts. The binary sequences used in the example correspond to the CZP case with S=2 and L=9 from Table 1.

Figure 1 depicts a design example for a Cantor ZP with S=2 and L=9 for both the O-CZP (opaque central region) and T-CZP (transparent central region) cases. The top image corresponds to the distribution of the Fresnel regions for a binary sequence length of L=9, which is common to both O-CZP and T-ZCP cases. Below, the ZP design procedure can be followed for each case. The left side corresponds to the O-CZP case, whereas the T-CZP case is illustrated in the right side. In each case, the corresponding binary sequence is stated, and the activation of opaque and transparent zones is illustrated below. Opaque Fresnel regions are depicted in white, while black is used to represent transparent Fresnel regions. As stated before, the example shown in Figure 1 corresponds to a CZP with S=2 and L=9, and its associated binary sequences can also be found in Table 1. Below the activation pattern, the corresponding pupil function, q(r), is depicted. The pupil function of a ZP indicates the transparency of each Fresnel region to the acoustic radiation. Thus, an opaque Fresnel region blocks the transmission of the acoustic wave (q(r)=0), whereas a transparent region allows the complete transmission of the acoustic radiation (q(r)=1). Finally, at the bottom of Figure 1, the ZP layout is depicted for both O-CZP and T-CZP cases. These layouts are generated by rotating the Fresnel activation pattern around the center of the lens.

### 2.2. Ultrasound Propagation with Directional Transducers

It is well known [1] that in the far field, a piston transducer placed at a distance *d* from the lens can be modelled as a point source emitter with a given directivity pattern. In this case, the incident pressure at the lens can be expressed as

(1) $$p_{i}\left(r\right)=\frac{jkp_{0}a^{2}}{2\sqrt{r^{2}+d^{2}}}D\left(r\right)e^{-jk\sqrt{r^{2}+d^{2}}},$$

where $p_{0}$ is the pressure at the piston surface, *a* is the piston active radius, *r* is the radial coordinate, *k* is the wavenumber, and D(r) is the piston directivity pattern, which is given by

(2) $$D\left(r\right)=\frac{2J_{1}\left(ka\frac{r}{\sqrt{r^{2}+d^{2}}}\right)}{ka\frac{r}{\sqrt{r^{2}+d^{2}}}},$$

being $J_{1}$ the first kind and first order Bessel function.

The incident power ($P_{i}$) on the lens plane and the transmitted power ($P_{t}$) throughout the lens are given by the following expressions,

(3) $$P_{i}=\frac{1}{2\rho_{0}c_{0}}{\;\int \int \;}_{S_{l}}{\left|p_{i}\left(r\right)\right|}^{2}dS$$

(4) $$P_{t}=\frac{1}{2\rho_{0}c_{0}}{\;\int \int \;}_{S_{l}}q\left(r\right){\left|p_{i}\left(r\right)\right|}^{2}dS$$

where $\rho_{0}$ is the medium density, $c_{0}$ is the speed of sound in the medium, $r_{N}$ is the outer radius of the lens, and q(r) is the pupil function of the lens as previously stated. This pupil function describes the geometry of the lens, indicating whether a specific region is transparent or opaque to the ultrasound emission by means of its binary sequence. In SZPs, the pupil function is 1 at the transparent regions and 0 at the opaque regions, that is, the oppose of the values indicated by the corresponding binary sequence. The calculation of the radii of the different Fresnel regions is required to implement this pupil function q(r). These radii can be obtained from the expression used to design SZPs with source point excitation,

(5) $$d+F+\frac{n\lambda }{2}=\sqrt{r_{n}^{2}+d^{2}}+\sqrt{r_{n}^{2}+F^{2}}.$$

where *F* is the focal length, *d* is the distance between the piston transducer and the SZP, λ is the wavelength, and $r_{n}$ stands for the external radius of each Fresnel region, with n=1,2,…,N, being *N* the total number of Fresnel regions of the SZP. This Equation is easily derived from the Fresnel zone construction principle [15], which establishes that the path difference between the direct path and the diffracted path through a Fresnel zone must be an integer multiple of λ/2.

**Figure 2:**
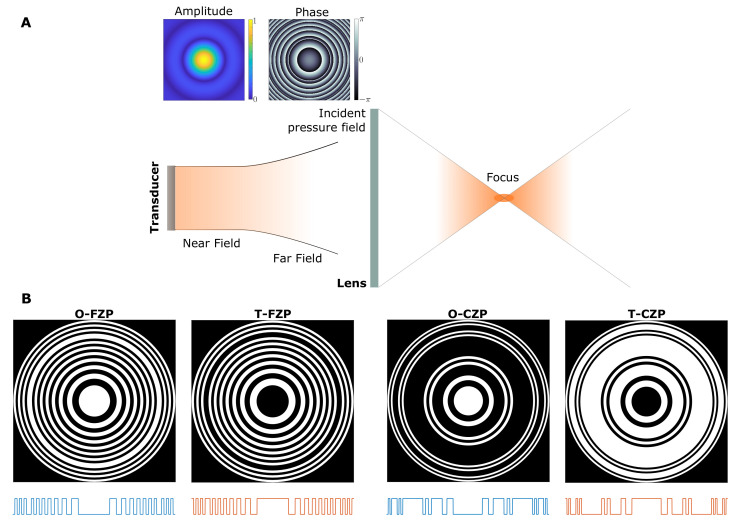
Schematic view of the system. (**A**) A piston transducer placed at a distance *d* from the lens, in its far field region, generates an incident wavefront influenced by the directivity of the transducer, as shown in the amplitude and phase insets. (**B**) Layouts of the different cases considered in this work, with their corresponding pupil functions shown below each layout.

Figure 2A depicts the schematic of the focusing procedure with a SZP and a directional transducer, with the insets showing the amplitude and phase of the incident pressure field generated by the transducer in the lens plane. Figure 2B shows different SZP layouts corresponding to the O-FZP, T-FZP, O-CZP and T-CZP cases. Below each layout, the pupil function of the corresponding SZP is depicted, illustrating the distribution of transparent and opaque Fresnel regions in each case. The FZP examples are shown for a binary sequence length of L=21 and they incorporate a phase correction ring (PCR) in order to mitigate the error introduced by the piston emitter as shown in [17]. The PCR is required when the lens is illuminated not only by the main lobe of the piston directivity pattern, but also by the adjacent secondary lobes. Thus, in order to counteract the π-phase shift introduced by the secondary lobe, it is necessary to shift outwards the activation of the Fresnel regions by just one region, but only in the area illuminated by the secondary lobe. This shift is achieved by inverting the activation of the Fresnel regions in this area. This phenomenon is critical when working with directional transducers and should be always considered during the design procedure. The use of PCRs in the design of ZPs is well explained and demonstrated in [17]. The CZPs examples are depicted for L=27 and S=3. The corresponding binary sequences can be found in Table 1. In this case, the use of PCRs is not required as the whole area of the CZP is illuminated by the main lobe of the piston directivity pattern.

In order to compare the lens efficiency in both T-SZP and O-SZP cases and be able to take the right design choice, two efficiency parameters are defined to characterize the focusing performance of the lens: the transmission enhancement factor ($\Delta \eta_{tx}$) and the focusing enhancement factor ($\Delta I_{F}$).

### 2.3. Transmission Enhancement Factor

The transmission efficiency of a lens, $\eta_{tx}$, is defined as the ratio between the transmitted power through the lens transparent zones and the total incident power at the lens, quantifying how much of the incident power crosses the lens. Thus, combining Equations (3) and (4), and using Equations (1) and (2), the lens transmission efficiency is given by

(6) $$\eta_{tx}=\frac{P_{t}}{P_{i}}=\frac{\int_{0}^{r_{N}}q\left(r\right)\frac{1}{r}J_{1}{\left(ka\frac{r}{\sqrt{r^{2}+d^{2}}}\right)}^{2}dr}{\int_{0}^{r_{N}}\frac{1}{r}J_{1}{\left(ka\frac{r}{\sqrt{r^{2}+d^{2}}}\right)}^{2}dr}.$$

In order to determine which type of SZP presents a higher transmission efficiency, either the T-SZP or the O-SZP, the Transmission enhancement factor is defined as the ratio between the transmission efficiencies for the T-SZP and O-SZP cases, and is given by

(7) $$\Delta \eta_{tx}=\frac{\eta_{tx}^{t}}{\eta_{tx}^{o}}=\frac{\int_{0}^{r_{N}}q^{t}\left(r\right)\frac{1}{r}J_{1}{\left(ka\frac{r}{\sqrt{r^{2}+d^{2}}}\right)}^{2}dr}{\int_{0}^{r_{N}}q^{o}\left(r\right)\frac{1}{r}J_{1}{\left(ka\frac{r}{\sqrt{r^{2}+d^{2}}}\right)}^{2}dr},$$

being $\eta_{tx}^{t}$ and $\eta_{tx}^{o}$ the transmission efficiencies for the T-SZP and O-SZP, respectively. Similarly, $q{\left(r\right)}^{t}$ and $q{\left(r\right)}^{o}$ correspond to the pupil functions for the T-SZP and O-SZP cases.

Therefore, $\Delta \eta_{tx}>1$ indicates that the T-SZP has the better transmission efficiency, whereas $\Delta \eta_{tx}<1$ results in a better performance on transmission efficiency for the O-SZP.

### 2.4. Focusing Enhancement Factor

Not only the transmission efficiency is important in the performance of the SZP. Another critical parameter is the maximum intensity value that the lens is able to achieve at its focus.

The acoustic intensity along the z-axis is given by [31],

(8) $$I\left(z\right)=\frac{4\pi^{2}}{\lambda^{2}}{\left|\int_{0}^{r_{N}}q\left(r\right)p_{i}\left(r\right)\frac{e^{-jk\sqrt{r^{2}+z^{2}}}}{\sqrt{r^{2}+z^{2}}}\frac{z}{\sqrt{r^{2}+z^{2}}}rdr\right|}^{2}$$

If we particularize Equation (8) at z=F, that is the intensity at the focus, we obtain:

(9) $$I_{F}=\frac{4\pi^{2}}{\lambda^{2}}{\left|\int_{0}^{r_{N}}q\left(r\right)p_{i}\left(r\right)\frac{e^{-jk\sqrt{r^{2}+F^{2}}}}{r^{2}+F^{2}}Frdr\right|}^{2}$$

Furthermore, combining Equations (1), (2) and (9),

(10) $$I_{F}=\frac{4\pi^{2}}{\lambda^{2}}{\left|\int_{0}^{r_{N}}q\left(r\right)\frac{jp_{0}aF}{r^{2}+F^{2}}J_{1}\left(ka\frac{r}{\sqrt{r^{2}+d^{2}}}\right)e^{-jk\sqrt{r^{2}+F^{2}}}dr\right|}^{2}$$

The focusing enhancement factor can be found as,

(11) $$\Delta I_{F}=\frac{I_{F}^{t}}{I_{F}^{o}}$$

being $I_{F}^{t}$ and $I_{F}^{o}$ the intensities at the focus for T-SZPs and O-SZPs, respectively.

Thus, combining Equations (10) and (11), the following final expression is obtained:

(12) $$\Delta I_{F}=\frac{{\left|\int_{0}^{r_{N}}q^{t}\left(r\right)J_{1}\left(ka\frac{r}{\sqrt{r^{2}+d^{2}}}\right)\frac{e^{-jk\sqrt{r^{2}+F^{2}}}}{r^{2}+F^{2}}dr\right|}^{2}}{{\left|\int_{0}^{r_{N}}q^{o}\left(r\right)J_{1}\left(ka\frac{r}{\sqrt{r^{2}+d^{2}}}\right)\frac{e^{-jk\sqrt{r^{2}+F^{2}}}}{r^{2}+F^{2}}dr\right|}^{2}}$$

Thus, $\Delta I_{F}>1$ indicates that the T-SZP focalizes better than the O-SZP, whereas if $\Delta I_{F}<1$, the O-SZP becomes the best design choice.

## 3. Simulation Results

Transmission efficiencies and focusing profiles have been calculated in MATLAB (The MathWorks Inc., Natick, MA, USA) using the analytical model described in the previous section. The parameters used in the simulation have been selected accordingly to the experimental measurements that are shown in the next section to improve the coherence and readability of the paper. Therefore, FZP lenses have been design with a binary sequence length L=31, an operating frequency f=270 kHz and a focal length F=80 mm, whereas the corresponding parameters for the CZP lenses are L=9, f=1 MHz and F=50 mm, respectively. The separation between the directional transducer and the SZP lens is the same for both cases, d=340 mm, although the piston transducer has a different active radius, a=15 mm for the FZP case and a=6.35 mm in the CZP simulations.

**Figure 3:**
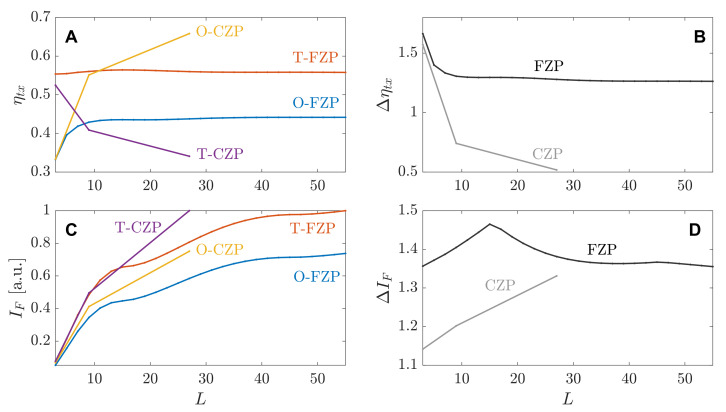
Simulation results of the focusing efficiency for the two considered lens types. (**A**) Transmission efficiency, (**B**) transmission enhancement factor, (**C**) normalized focal intensities, and (**D**) focusing enhancement factor. All results are given as a function of the binary sequence length.

Figure 3A,C depict the transmission efficiency ($\eta_{tx}$) and the intensity at the focus ($I_{F}$), respectively, against the length of the binary sequence for four different SZPs. $\eta_{tx}$ is dimensionless, while $I_{F}$ is represented in arbitrary units and normalized to the highest value in both the FZP and the CZP cases. The FZP curves are very smooth, as the length of the binary sequence is increased in steps of two units between simulation points. On the contrary, when considering the Cantor ternary set, a discrete number of binary sequences can be generated. In the binary sequence length span represented in the different subplots, only three binary sequences of fractal orders S=1, S=2, and S=3, can be represented. These binary sequences have lengths L=3, L=9 and L=27, respectively, and can be found in Table 1. The next fractal order, S=4 corresponds to a binary sequence of length L=81, which is out of the span represented in the figure. Thus, the curves corresponding to CZPs are more abrupt, as they only have three simulation points. Figure 3B,D depict the transmission enhancement factor ($\Delta \eta_{tx}$) and the focusing enhancement factor ($\Delta I_{F}$), respectively, against the length of the binary sequence for both the FZP and the CZP cases. Both $\Delta \eta_{tx}$ and $\Delta I_{F}$ parameters are dimensionless, and the CZP curves still represent only three simulation points, as explained above.

As can be observed from Figure 3A, in the conventional FZP case, the transmission efficiency is always higher when the central Fresnel region is transparent (T-FZP). As the length of the binary sequence increases, $\eta_{tx}$ tends steadily to 0.56 and 0.44 for the T-FZP and O-FZP cases, respectively. This behaviour is expected as the FZP has approximately the same number of transparent and opaque regions regardless of the length of the binary sequence. However, as it was pointed out when the analytical model was presented, CZPs have a more uneven distribution of transparent and opaque regions as can be verified from Table 1. Thus, in the two cases where the binary sequence length becomes significant, L=9 and L=27, there is a larger amount of transparent areas in the O-CZP than in the T-CZP, and consequently, the transmission efficiency is higher in the O-CZP case. For instance, $\eta_{tx}=0.34$ in the T-CZP case while $\eta_{tx}=0.66$ in the O-CZP case for L=27. Thus, as can be observed from Figure 3B the transmission enhancement factor for both FZP and CZP is completely different. While $\Delta \eta_{tx}$ is always higher than 1 in the FZP case (tends to a value of $\Delta \eta_{tx}=1.26$), $\Delta \eta_{tx}<1$ for the two binary sequences of significant length (L=9 and L=27) in the CZP case, which is due to the fact that there is more power transmitted though the O-CZP lens than through the T-CZP lens. The lower transmission enhancement factor corresponds to a value of $\Delta \eta_{tx}=0.52$ for L=27. One could deduce that the focusing enhancement factor was going to behave in a similar way, but surprisingly this is not the case. As can be observed from Figure 3C, the intensity at the focus is always higher in the T-SZP case than in the O-SZP case, regardless of considering either FZPs or CZPs. This translates in $\Delta I_{F}$ values higher than 1 for both the FZP and CZP cases, as can be observed from Figure 3D. The maximum $I_{F}$ values are 1, 0.74, 1 and 0.75 for the T-FZP, O-FZP, T-CZP and O-CZP cases, respectively. It is worth noting that the $I_{F}$ parameter is normalized to the maximum value, that corresponds to the T-SZP lens, for both FZP and CZP cases. Figure 3D shows a peak value of $\Delta I_{F}=1.47$ for the FZP lens, although the tendency after the peak is decreasing, reaching a value of $\Delta I_{F}=1.36$ for L=55, while the maximum value in the CZP case is $\Delta I_{F}=1.33$ for L=27. It is noticeable that in the CZP case, not only is the focusing enhancement factor higher than 1 when, in fact, there is less transmitted power through the T-CZP lens, but also the trend of the $\Delta I_{F}$ curve is clearly upwards, while the trend of the $\Delta \eta_{tx}$ curve is downwards in the opposite direction. Therefore, when *L* is increased in the CZP case, the transmission enhancement factor becomes smaller while the focusing enhancement factor increases its value. The explanation of this unexpected behaviour has to be understood from the nature of the different parameters. When dealing with $\Delta \eta_{tx}$, the raw power that crosses the SZP is considered. On the other hand, the $\Delta I_{F}$ parameter is calculated from focusing profiles that are formed as a result of the interference of the pressure acoustic field waves. Moreover, the spot is directed to a particular location, that of the main focus. Thus, a T-SZP lens, even in the cases where it transmits less raw power, becomes the optimum choice for the implementation of a SZP lens. Additional simulations have been carried out for other types of SZPs with different binary sequences, and the result is always the same. The optimum implementation choice is the T-SZP lens, regardless of the transmission efficiency of the lens. Thus, achieving a higher focusing efficiency can be directly related to the transparency of the central Fresnel region, which in turn allows the direct path between the ultrasound emitter and the focus.

## 4. Experimental Measurements

Several experimental measurements have been carried out to demonstrate the focusing enhancement achieved combining T-SZPs with directional transducers. The experimental set-up consists of a 3D underwater automated positioning system with a spatial resolution of 1×1×1 mm${}^{3}$ and three degrees of freedom. The signal is generated using a Panametrics 5077PR pulser and then, it is transmitted using a piston transducer. A needle hydrophone from Precision Acoustics with a diameter of 1.5 mm is used as receiver, and its output signal is amplified using a low noise preamplifier, and then digitized using a digital oscilloscope from Pico Technology. A piston transducer from Imasonic with a 12.7 mm active diameter and a central frequency of 1 MHz is used to characterize the CZPs, whereas a different piston transducer also from Imasonic with a 30 mm active diameter and a central frequency of 260.88 kHz is used in the FZP measurements. This is mainly due to the availability of the emitters, as the measurements were performed at different times. As a consequence of the transducer selection, some other design parameters, such as the lens working frequency (*f*) or the focal length (*F*) are also different. All FZPs and CZPs have been manufactured using brass for implementing the opaque Fresnel regions, because this material presents a high acoustic impedance mismatch with water. CZPs have been designed using the binary sequences associated to the Cantor ternary set with S=2 for an operating frequency of 1 MHz and, a binary sequence of length L=9, and a focal distance F=50 mm, while FZPs have been designed for an operating frequency of 250 kHz, a binary sequence of length L=31, and a focal distance F=80 mm. The separation between the lens and the transducer is the same in both measurements, d=340 mm.

**Figure 4:**
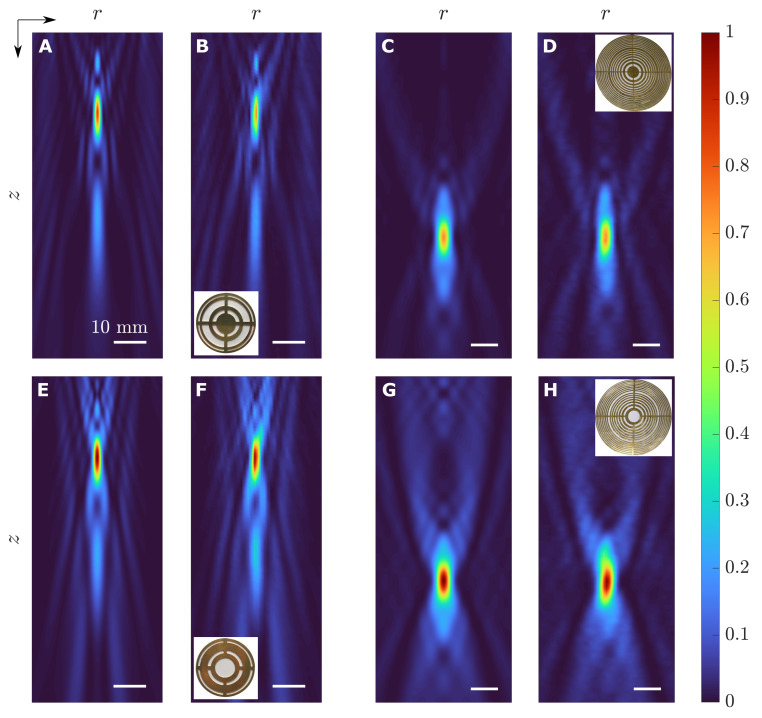
Intensity maps for the O-CZP case: (**A**) simulated and (**B**) experimental; O-FZP case: (**C**) simulated and (**D**) experimental; T-CZP case: (**E**) simulated and (**F**) experimental; and T-FZP case: (**G**) simulated and (**H**) experimental. Each experimental intensity map shows an inset with an image of the corresponding manufactured lens. All maps have been normalized to the maximum intensity values, corresponding to the cases with the transparent central region.

**Figure 5:**
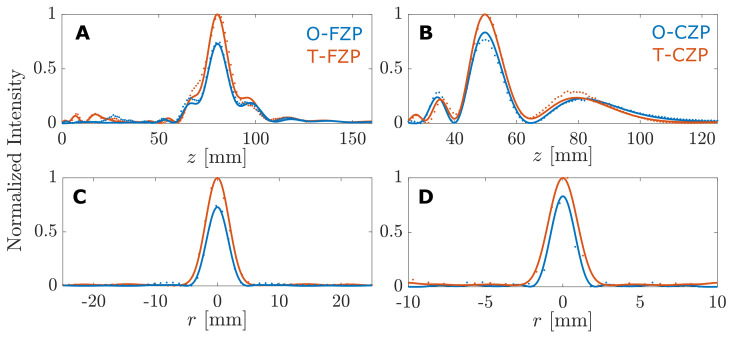
(**A**,**B**) Longitudinal and (**C**,**D**) lateral intensity cuts from the maps depicted in Figure 4. (**A**,**C**) correspond to the FZP case, while (**B**,**D**) correspond to the CZP lens. Simulation and experimental results are depicted with solid and dotted lines, respectively.

Figure 4 depicts the simulated and measured intensity maps for the considered FZP and CZP cases. The first and the third columns of the figure correspond to numerical simulations, whereas the second and the fourth columns represent experimental measurements. As can be observed, there is an excellent agreement between numerical intensity calculations and experimental results in all four lens cases. Moreover, as can be observed from the intensity maps of Figure 4, the intensity at the focus is higher in the T-SZP case as expected, regardless of whether a FZP or a CZP is considered. Figure 5 represents the longitudinal (r=0) and radial (z=F) cuts of the intensity maps shown in Figure 4, which correspond to the axial focusing profiles and lateral profiles, respectively. Solid lines are used to depict simulation results, while dotted lines are used to depict the experimental measurements, and T-SZPs and O-SZPs have been depicted in blue and red, respectively. The intensity is normalized to the maximum value, which always corresponds to the T-SZP case. The focusing and lateral profiles of Figure 5 show in detail that the simulation results match the experimental measurements very well.

Some of the main parameters of experimental measurements have been summed up in Table 3. *f* is the ultrasound frequency used in each experiment. *F* represents the experimental focal length and matches perfectly the design parameter. FLHM and FWHM stand for the Full Length Half Maximum and the Full Width Half Maximum, respectively. The FLHM parameter corresponds to the “Length” of the focus in the axial direction, and it is calculated from the difference between the *z* locations, at opposite sides of the focus central point, at which the normalized intensity is half of its maximum value. Analogously, the FWHM represents the width of the focus along the lateral direction. Both FLHM and FWHM parameters are related to the longitudinal and lateral resolutions, respectively. As the value of any parameter is diminished, the corresponding resolution increases and the focus becomes slimmer. $I_{F}$ is the measured peak intensity at the focus and is represented in arbitrary units, normalized to the maximum value, which corresponds to the T-SZP case. As can be observed from Table 3, although T-SZPs achieve a higher intensity at the focus, they present a decrease in lateral resolution (higher FWHM) in both FZP and CZP cases. The lens longitudinal resolution is also reduced in the FZP case (higher FLHM value for the T-FZP lens), although it remains steady in the CZP case, where very similar FLHM values are achieved for both the O-CZP and the T-CZP cases. Moreover, as can be seen in Table 3, CZPs achieve higher longitudinal and lateral resolutions than FZPs. This is mainly due to the difference in the operating frequencies of both devices. In terms of the wavelength, the resolutions for the CZP are approximately FLHM≈7.6λ and FWHM≈1.2λ, while FLHM≈2.34λ and FWHM≈0.72λ correspond to the FZP case. Therefore, FZPs achieve higher resolutions than CZPs, as expected, due to the difference between the number of Fresnel regions in each scenario (31 for the FZP case and 9 for the CZP case).

Table 4 shows the simulation and experimental focusing enhancement parameters for both FZP and CZP cases. Again, the agreement between simulation and experimental measurements is very good, which validates the analytical model presented in this paper. The focusing enhancement is slightly higher in the CZP case, although this result is not very significant as the CZP and FZP lenses are not directly comparable, because they have been measured with different transducers at distinct ultrasound frequencies and focusing at different focal lengths. The result that is really significant from this table is the fact that the experimental measures confirm that the CZP lens with L=9 presents a $\Delta I_{F}>1$, specifically $\Delta I_{F}=1.35$, that translates into a better focusing performance for the T-CZP lens, although as it was pointed out in the simulation for this particular case, the O-CZP lens presents a much better transmission efficiency than the T-CZP lens.

## 5. Conclusions

In this work, we have analyzed the impact that the distribution of transparent and opaque Fresnel regions has on the focusing capability of a SZP generated from a binary sequence, when considering directional emitters. An analytical model that introduces two efficiency parameters in order to determine the optimum distribution for the Fresnel regions has been developed. These efficiency parameters are the transmission enhancement factor ($\Delta \eta_{tx}$) and the focusing enhancement factor ($\Delta I_{F}$). Simulations have shown that, although at first sight the transmission efficiency ($\eta_{tx}$) of the SZP should be an important parameter in determining the selection of the optimum SZP, it has no weight on the decision. The key parameter is $\Delta I_{F}$, and the right decision is always to take the T-SZP approach, because $\Delta I_{F}$ is always higher than 1. It has been shown that a CZP based on a binary sequence of length L=27 provides a $\Delta \eta_{tx}$ as low as 0.5, but results in a focusing enhancement factor of $\Delta I_{F}=1.35$, that is the improvement of the T-CZP over the O-CZP lens is over 35%, although the transmission efficiency of the T-CZP lens is half of the O-CZP value. Simulation results have been validated with experimental measurements for both FZP and the CZP lenses. This validation proves the feasibility of the analytical model and provides an easy design decision when a SZP is required in applications with directional transducers.

We have demonstrated that no matter the type of ZP lens that is being employed (FZPs, CZPs or any other kind of SZP based on binary sequences), the best alternative is always the transparent case (T-SZP), with independence of the transmission efficiency. As an example, we have shown ZPs based on Cantor binary sequences, where a higher transmission efficiency is obtained in the opaque scenario (O-CZP), but the higher focusing peak is achieved again at the T-CZP case, contrary to what initially could be expected. This behaviour is replicated for any other ZP based on a different binary sequence regardless of the transmission enhancement factor. Therefore, the transmission efficiency is not a critical parameter that should be considered for taking the design decision of selecting an opaque or a transparent central Fresnel region in order to improve the ZP focusing profile, and the right choice is always to go with the transparent central area (T-SZP), regardless of the type of ZP lens or its transmission efficiency. This is an important issue that should be widespread, as many researchers still manufacture their ZPs with an opaque central region (O-SZP), reducing the dynamic range of their ZP focusing profiles and applications.

## Figures and Tables

**Table 1:** Binary sequences for different CZPs and their associated FZPs.

*S*	*L*	Zone Plate	Binary Sequence
1	3	O-CZP	{101}
		T-CZP	{010}
		O-FZP	{101}
		T-FZP	{010}
2	9	O-CZP	{101000101}
		T-CZP	{010111010}
		O-FZP	{101010101}
		T-FZP	{010101010}
3	27	O-CZP	{101000101000000000101000101}
		T-CZP	{010111010111111111010111010}
		O-FZP	{101010101010101010101010101}
		T-FZP	{010101010101010101010101010}

**Table 2:** Binary sequence examples for different types of SZP.

Zone Plate	*L*	Binary Sequence
Fibonacci SZP	21	{101101011011010110101}
Predistorted SZP	19	{1111111010101010101}
Thue–Morse SZP	16	{1001011001101001}

**Table 3:** Measured FLHM, FWHM and normalized focal intensity values for the SZPs depicted in Figure 4 and Figure 5.

	f (kHz)	F (mm)	FLHM (mm)	FWHM (mm)	$I_{F}\;[a.u.]$
O-FZP	270	80	12.85	3.86	0.77
T-FZP	270	80	14.45	4.34	1.00
O-CZP	1000	50	11.48	1.59	0.74
T-CZP	1000	50	11.39	2.09	1.00

**Table 4:** Simulated and experimental focusing enhancement factors for both FZP and CZP cases.

	$\Delta I_{F}^{sim}$	$\Delta I_{F}^{exp}$
FZP	1.20	1.30
CZP	1.37	1.35

## Data Availability

Data will be available upon reasonable request to the corresponding author.
